# Delirium in Internal Medicine Departments in a Tertiary Hospital in Israel: Occurrence, Detection Rates, Risk Factors, and Outcomes

**DOI:** 10.3389/fmed.2020.581069

**Published:** 2020-10-19

**Authors:** Nirit Tzur Efraim, Evgeniya Zikrin, David Shacham, Dori Katz, Evgeni Makulin, Leonid Barski, Lior Zeller, Carmi Bartal, Tamar Freud, Svetlana Lebedinski, Yan Press

**Affiliations:** ^1^Department of Psychiatry, Soroka Medical Center, Beer-Sheva, Israel; ^2^Faculty of Health Sciences, Ben-Gurion University of the Negev, Beer-Sheva, Israel; ^3^Department of Geriatrics, Soroka Medical Center, Beer-Sheva, Israel; ^4^Department of Internal Medicine F, Soroka Medical Center, Beer-Sheva, Israel; ^5^Department of Internal Medicine C, Soroka Medical Center, Beer-Sheva, Israel; ^6^Department of Internal Medicine E, Soroka Medical Center, Beer-Sheva, Israel; ^7^Faculty of Health Sciences, Siaal Research Center for Family Medicine and Primary Care, Ben-Gurion University of the Negev, Beer-Sheva, Israel; ^8^Clinical Pharmacy Services, Pharmacy Department, Clalit Health Services, Beer-Sheva, Israel; ^9^Unit for Community Geriatrics, Division of Health in the Community, Ben-Gurion University of the Negev, Beer-Sheva, Israel; ^10^Center for Multidisciplinary Research in Aging, Ben-Gurion University of the Negev, Beer-Sheva, Israel

**Keywords:** delirium, occurence, risk facors, detection rate, outcomes

## Abstract

**Background:** The aim of the study was to assess the occurrence rate of delirium among elderly hospitalized patients in the medicine wards of a large tertiary hospital, to identify risk factors, and to evaluate the diagnostic rate for delirium among the medical teams.

**Methods:** A 3-month prospective study of patients 65 years of age and above in three medicine wards: in two wards patients were examined by trained study team members using the Confusion Assessment Method (CAM), while the third was a control ward where CAM was not administered. The third ward served to control for the effect of the presence of investigators in the other wards as a potential confounding factor. Based on the results of this assessment patients were defined as suffering from *subsyndromal delirium, full delirium* (these two groups were later combined into an “*any symptoms of delirium”* group), and *no delirium*. The rate of diagnosis by the medical team was obtained from the electronic medical records.

**Results:** The full delirium rate was 5.1%, the rate of subsyndromal delirium was 14.6%, and the rate of any symptoms of delirium was 19.6%. Absence of a partner, pain, anemia, hyponatremia, hypocalcemia, and the use of drugs with an anticholinergic burden were factors for any symptoms of delirium as well as for subsyndromal delirium. Subsyndromal delirium and any symptoms of delirium were associated with a reduced chance of being discharged to home and a higher 3-month mortality rate. A diagnosis of delirium was found in only 19.4% of the patients with any symptoms of delirium in the medical records.

**Conclusions:** Delirium is a common problem among elderly hospitalized patients, but it is diagnosed sub-optimally by the medical team. There is a need for further training of the medical teams and implementation of delirium assessment as part of the ward's routine.

## Introduction

Delirium is a neurocognitive disorder characterized by disturbance of attention or awareness, accompanied by a change in baseline cognition that cannot be explained by a preexisting or evolving neurocognitive disorder. The disturbance develops over a short period of time and tends to fluctuate over the course of the day (1).

Delirium is a highly prevalent problem with an occurrence rate of 7–42% among adults hospitalized in internal medicine wards (2–5) and is associated with poor outcomes, including an increased risk of dementia and functional decline (6), prolonged hospital stay (5, 7, 8), increased risk of institutionalization (3, 6, 7), high health care costs (9), and even increased mortality (3, 5, 6, 10, 11). Despite its clinical significance delirium often remains undiagnosed by the medical team. The rate of diagnosis by doctors has been reported as 7–57% (12–15) and by nurses 26–83% (16–18).

In Israel only a limited number of studies have assessed the occurrence, risk factors, and outcomes for delirium in the elderly population. These studies were conducted among patients with femoral fractures (19, 20), patients in the geriatrics wards (21) and patients in the long-term care hospitals (22). To our knowledge there is no report of prospective studies on the occurrence of delirium among elderly patients hospitalized in medicine wards of a general hospital in Israel. Thus, the aim of the study was to assess the occurrence rate, the diagnostic rate by the medical team, risk factors, and health outcomes for delirium in this patient population.

## Methods

The study was conducted in the Soroka University Medical Center, a tertiary hospital with 1,151 beds. It is the third largest tertiary care center in Israel and the largest in southern Israel. It's Division of Internal Medicine consists of seven Internal Medicine wards and a Geriatrics Department. The study was conducted in three internal medicine wards by a team of investigators that included residents in psychiatry and geriatrics, and experts in internal medicine and geriatrics.

### Study Population

The study population included men and women 65 years of age and over who were hospitalized in one of the study wards for any reason, and who spoke Hebrew, Russian, or English. Exclusion criteria included patients who were unconscious, ventilated, whose medical condition necessitated intervention and could not be assessed, who suffered from known cognitive impairment to a degree that made verbal communication impossible, and stroke patients who could not communicate verbally.

### Study Protocol

The study was conducted over a 3-month period from December 15, 2019 to March 14, 2020. Prior to the start of the study the investigators held a meeting with the medical teams in the three wards. At the meeting they presented the aims of the study and gave a 30-min lecture on delirium and ways to prevent it. The team members received a printed card with the means of preventing delirium for their personal use and to distribute to patients and their families.

Delirium was evaluated using the Confusion Assessment Method (CAM) (23). Data on acute onset and fluctuating course were collected from the medical team or from the patient's family. Over the 3-month study follow-up, the research team conducted a daily visit to two of the three wards (CAM wards) and conducted a delirium assessment at least once for patients who met the inclusion criteria. Based on the study plan we intended to assess all patients in the CAM group in Ward A on a daily basis, except for weekends, while in the CAM group in Ward B we planned to assess them once, at or close in time to admission to the ward. This plan was designed to answer the question: Does a high frequency of assessment increase the diagnostic rate for delirium in light of the fluctuating nature of the disorder?

The initial assessment was conducted on the day after hospitalization unless the patient was hospitalized on the weekend, in which case it was conducted on the first weekday. Several independent doctors on the two CAM wards were appointed to go over the lists of the newly hospitalized patients and identify patients who met the study inclusion criteria. Assessments was conducted between 10 A.M. and 3 P.M. to enable the independent doctors to get to know the new patients and decide if they were met the study inclusion criteria. When the independent doctors confirmed a patient for the study the investigators approached the patient, explained the study aims to them and asked for oral consent to undergo the CAM test. *After the CAM test, patients without delirium signed an informed consent form and were included in the study. The patients with delirium were asked to sign informed consent at a later date, when their state of delirium had passed. In accordance with the decision of the Helsinki Committee, patients who were not able to sign informed consent at any point were also included in the data analyses*. Each case of delirium that was diagnosed over the course of the study was reported personally to the ward's medical staff and documented in the patient's electronic medical record (EMR). In the third ward (control ward) we did not conduct CAM assessments. This was designed to help us understand the extent to which the presence of the research team in the ward affected the diagnosis of delirium by the medical team. In this ward a diagnosis of delirium was made by the ward staff without the use of CAM.

The following data were collected from the EMR:

- Socio-demographic data—age, sex, family status, living conditions, nursing care.
- Clinical and laboratory data—diseases leading to hospitalization, co-morbid diseases. The Charlson comorbidity index (CCI) was calculated (24), the list of regular medications given in the community and those given over the course of hospitalization, the Anticholinergic Burden (ACB) (25) (which was calculated), vital signs throughout hospitalization, and the results of laboratory tests at admission to the hospital.
- Hospitalization outcome—discharge from the hospital (to the community or long-term care), death, transfer to another hospital, and death over the first 90 days after discharge.

Based on the results of the CAM test the patients were classified into four groups:

1. Full delirium—when two core criteria were met (acute onset and fluctuating course, and inattention) and at least one of altered level of attention and disorganized thinking.
2. Subsyndromal delirium (SSD)—when one of the core criteria was met and at least one of altered level of consciousness or disorganized thinking.
3. Any symptoms of delirium (ASD)—all cases of full delirium and SSD.
4. No delirium

### Statistical Analyse

Statistical analyses were performed using the IBM SPSS Statistical software version 26 (SPSS, Inc, Chicago, IL). A *P* < 0.05 was considered statistically significant. The demographic and clinical characteristics of patients with and without delirium were compared. Categorical variables are described as frequencies and percentiles. Continuous variables, such as age, are described as mean ± standard deviation. The analysis of specific risk factors associated with delirium was carried out using univariate analysis. Differences in categorical variables were tested by the Chi-square or the Mid-P exact tests, in accordance with the size of the cells. The Mann–Whitney *U*-test, or the Student *t*-test, was used to compare continuous data depending on the data distribution.

The study was approved by the Helsinki Committee of the Soroka Medical Center (SOR-0047-19) and the Ministry of Health Committee (Form 8, Request #201911315, dated November 4, 2019).

## Results

**Figure 1:**
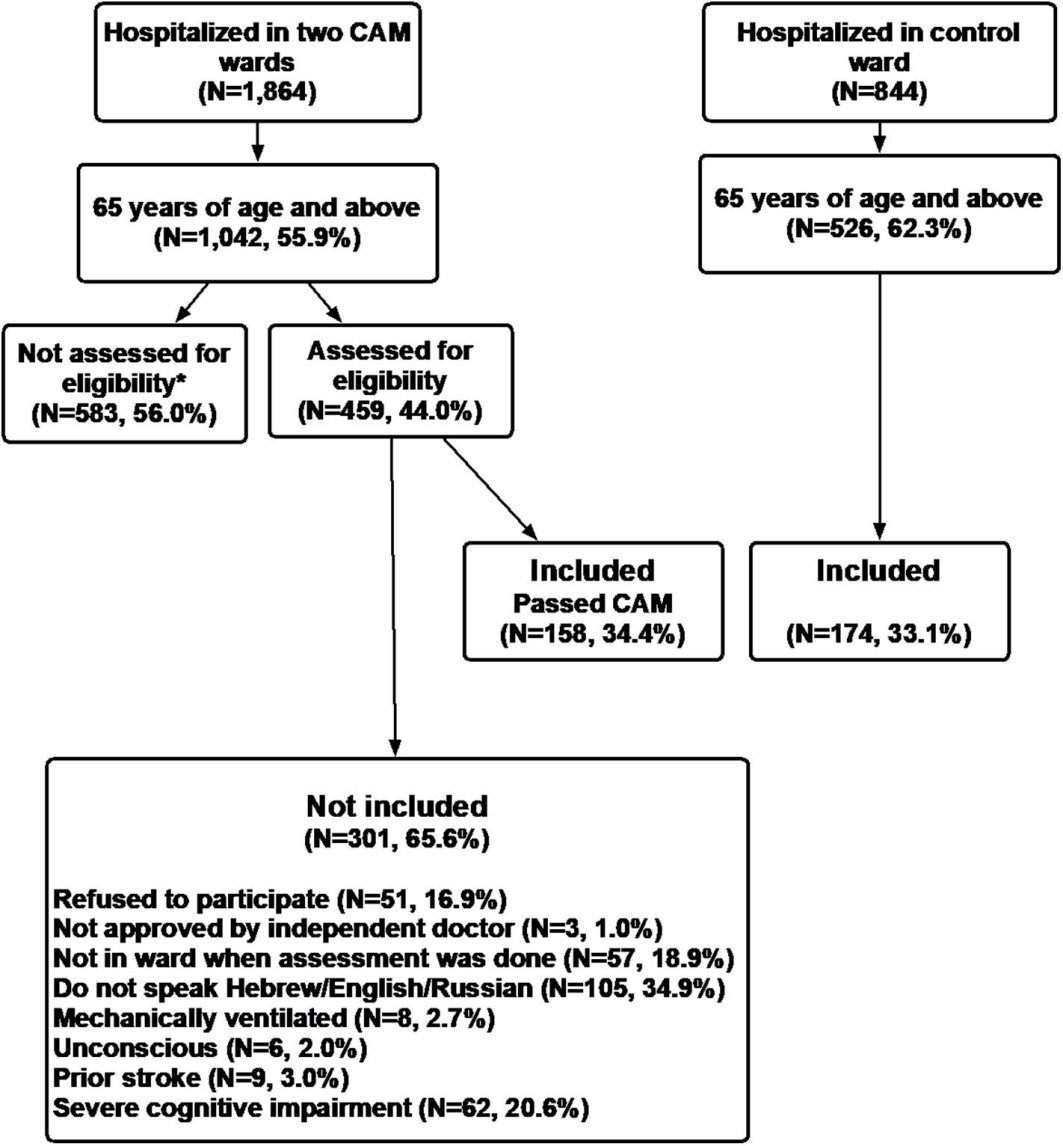
*Not assessed for eligibility: although the patients met the inclusion criteria there was no independent doctor or research doctor available on some of the days, and cessation of recruitment due to the Coronavirus outbreak–one of the two CAM wards was closed and converted to a Covid-19 ward.

Over the study period (December 15, 2019 to March 14, 2020) 1,864 patients were hospitalized in the two CAM wards and 844 in the control ward. Of these, 1,042 (55.9%) were 65 years old or above in the CAM wards and 526 (62.3%) in the control ward. In all, 332 patients participated in the study. In the CAM wards 158 patients (15.1% of the eligible patients in those wards during the study period) underwent CAM test. In the control ward the medical records of 174 patients were reviewed (33% of the eligible patients in that ward), and on every day that patients were recruited into the study in the CAM wards a similar number of patients was recruited in the control ward (Figure 1).

### The Rate of Delirium in the CAM Group

In this group we conducted 229 CAM tests in 158 patients. All 158 patients underwent a CAM test at least once, with 38 of the 73 patients in Ward A undergoing CAM at least twice, 15 had three, 10 had four, and 7 had five. Eight patients (5.1%) in the CAM group had full delirium over the course of the study, 23 (14.6%) were classified as SSD (20 of 23 were lacking the acute onset and fluctuating course criterion). Among the eight cases of full delirium one had SSD at the first assessment and met the criteria for full delirium the next day. In another case of delirium there were no signs of delirium on the first assessment, but full delirium was diagnosed the next day on repeat assessment, in two other cases later assessments showed that delirium had diminished with only one criterion met. In all, 31 patients, or 19.6% of the 158 patients who underwent the assessment within the framework of the study were included in the ASD group. Five cases of full delirium were diagnosed among the 73 patients (6.9%) in Ward A and three (3.5%) were diagnosed among the 85 patients in Ward B (*P* = 0.473), SSD was found in 3 (3.5%) in Ward A and 20 (23.5%) in Ward B (*P* = 0.0005), in all there were 8 cases of ASD (11.0%) in Ward A 23 (27.1%) in Ward B (*P* = 0.015).

### Risk Factors for Delirium in the CAM Group

Because of the low number of cases with full delirium we analyzed risk factors of delirium in the SSD and ASD groups only.

#### SSD vs. No Delirium

The results of a comparison between the 23 patients with SSD and the 127 without delirium showed that those with SSD had a lower percentage of married patients (26.1 vs. 53.5%, *P* = 0.017), a higher mean ACB (2.7 ± 1.5 vs. 1.6 ± 1.5, *P* = 0.0013; Table 1), a lower hemoglobin level at admission (10.8 ± 2.1 vs. 12.2 ± 2.3, *P* = 0.008), higher levels of urea and creatinine at admission (2.1 ± 2.2 vs. 1.2 ± 1.0, *P* = 0.001 and 85.9 ± 88.5 vs. 54.1 ± 33.8, *P* = 0.003, respectively), a higher percentage of hyponatremia at admission (30.4 vs. 10.2%, *P* = 0.02), a higher rate of hypocalcemia at admission (43.5 vs. 38.7%, *P* = 0.0001), a higher percentage of patients with bradycardia on the day of the CAM test (30.4 vs. 11.8%, *P* = 0.036), and a VAS pain score >3 (13.0 vs. 0.0%, *P* = 0.05; Table 2).

**Table 1:** Socio-demographic characteristics, comorbidity, medical treatment and reasons for hospitalization in the CAM group.

	**SSD**	**ASD**	**No D**	**P**	**P**
	***N* = 23**	***N* = 31**	***N* = 127**	**SSD vs. No D**	**ASD vs. No D**
Age, mean ± SD	76.3 ± 9.3	77.3 ± 9.4	74.9 ± 6.8	0.711	0.271
Gender (male), *N* (%)	11 (47.8)	16 (51.6)	71 (55.9)	0.484	0.671
Family status (married), *N* (%)	6 (26.1)	7 (22.6)	68 (53.5)	0.017	0.0019
Living status (alone), *N* (%)	7 (30.4)	9 (29.0)	38 (29.9)	0.945	0.938
Nursing caregiver (yes), *N* (%)	11 (47.8)	17 (54.8)	42 (33.1)	0.188	0.03
Charlson's comorbidity index (total score), mean ± SD	2.8 ± 1.7	2.6 ± 1.7	2.4 ± 1.8	0.232	0.394
Dementia per history, *N* (%)	3 (13.0)	7 (22.6)	5 (3.9)	0.125	0.003
Medications during hospitalization, mean ± SD	7.5 ± 2.7	7.4 ± 2.7	6.6 ± 3.1	0.153	0.144
AC burden, mean ± SD	2.7 ± 1.5	2.9 ± 1.6	1.6 ± 1.5	0.001	<0.0001
**Ds during hospitalization**, ***N*** **(%)**					
Any infection, *N* (%)	4 (17.4)	8 (25.8)	24 (18.9)	0.9	0.4
Cardio-vascular (IHD, HF, arrhythmia, CVA), *N* (%)	11 (47.8)	12 (38.7)	68 (53.5)	0.622	0.146
Gastro-intestinal problems, *N* (%)	3 (13.0)	4 (12.9)	5 (3.9)	0.125	0.09
COPD/Asthma, *N* (%)	3 (13.0)	4 (12.9)	14 (11.0)	0.752	0.748
Musculoskeletal problems, *N* (%)	5 (21.7)	5 (16.1)	16 (12.6)	0.271	0.56

*D, Delirium; ASD, any symptoms of delirium; SSD, Subclinical Delirium; AC, anticholinergic; COPD, chronic obstructive pulmonary disease; IHD, ischemic heart disease; HF, heart failure; CVA, cerebrovascular accident*.

**Table 2:** Clinical and laboratory characteristics of the CAM group [Number (%) unless otherwise stated].

	**SSD**	**ASD**	**No D**	**P**	**P**
	***N* = 23**	***N* = 31**	***N* = 127**	**SSD vs. No D**	**ASD vs. No D**
**Signs on the day of CAM**					
Temperature ≥38.0°C	0 (0.0)	1 (3.2)	4 (3.1)	0.671	0.932
Hypertension (Systolic BP ≥150 mmHg)	3 (13.0)	5 (16.1)	28 (22.0)	0.347	0.45
Hypotension (Systolic BP <90 mmHg)	1 (4.4)	1 (3.2)	1 (0.8)	0.307	0.392
Tachycardia (HR >100 per min)	1 (4.4)	2 (6.5)	4 (3.1)	0.739	0.425
Bradycardia (HR <60 per min)	7 (30.4)	7 (22.6)	15 (11.8)	0.036	0.145
Pain (VAS>3)	3 (13.0)	3 (9.7)	0 (0.0)	0.05	0.028
**Lab on admission**					
Hemoglobin (g/dl), mean ± SD	10.8 ± 2.1	11.1 ± 2.1	12.2 ± 2.3	0.008	0.011
WBC (x10^3^ uL), mean ± SD	7.9 ± 4.6	8.7 ± 4.7	9.8 ± 7.4	0.24	0.444
Glucose (mg/dl), mean ± SD	133.3 ± 54.9	137.7 ± 71.1	156.5 ± 79.3	0.189	0.074
Creatinine (mg/dl), mean ± SD	2.1 ± 2.2	1.9 ± 2.0	1.2 ± 1.0	0.001	0.063
Urea (mg/dl), mean ± SD	85.9 ± 88.5	83.6 ± 80.4	54.1 ± 33.8	0.003	0.059
C-reactive protein, mean ± SD	3.9 ± 5.4	4.9 ± 7.3	3.3 ± 5.6	0.624	0.068
Hyponatremia (Sodium <135 mEq/L)	7 (30.4)	8 (22.6)	13 (10.2)	0.02	0.036
Hypernatremia (Sodium>145 mEq/L)	0 (0.0)	0 (0.0)	3 (2.4)	0.53	0.488
Hypocalcemia (Calcium <8.5 mg/dl)	10 (43.5)	12 (38.7)	11 (8.7)	0.0001	0.002
Hypercalcemia (Ca >10.5 mg/dl)	0 (0.0)	0 (0.0)	2 (1.6)	0.382	0.488

*D, Delirium; ASD, any symptoms of delirium; SSD, Subclinical Delirium; BP, blood pressure; HR, heart rate; VAS, Visual Analog Scale; WBC, white blood cells*.

#### ASD vs. No Delirium

The results of a comparison between the 31 patients with ASD and the 127 patients without delirium showed that fewer in the ASD group were married (22.6 vs. 53.5%, *P* = 0.0019), more had a nursing caregiver (45.2 vs. 66.9%, *P* = 0.03), more a higher percentage of dementia (22.6 vs. 3.9%, *P* = 0.003), a higher mean ACB score (2.9 ± 1.6 vs. 1.6 ± 1.5, *P* < 0.0001; Table 1), a lower hemoglobin level on admission (11.1 ± 2.1 vs. 12.2 ± 2.1, *P* = 0.011), a higher percentage of hyponatremia on admission (22.6 vs. 10.2%, *P* = 0.036), and a higher percentage of hypocalcemia on admission (38.7 vs. 8.7%, *P* = 0.002). At the time of CAM administration, the patients in the ASD group had higher levels of pain (VAS>3) (9.7 vs. 0%, *P* = 0.028; Table 2).

To better understand the differences in rates of ASD and SSD between patients in the two experimental wards, we compared the patients' characteristics in these two wards in terms of socio-demographic data, and risk factors for the development of delirium in the entire CAM group (Table 3). The patients in Ward A were younger 74.5 ± 7.5 vs. 76.4 ± 7.2 years, had a higher percentage of men (63.0 vs. 48.2%), and a lower percentage of nursing assistance (21.9 vs. 36.5%), but none of these differences was statistically significant. No differences were found between groups in the other indices except for the percentage of married patients that was higher in Ward A (57.5 vs. 38.8%, respectively, *P* = 0.025).

**Table 3:** Basic characteristics of Wards A and B.

	**Ward A *N* = 73**	**Ward B *N* = 85**	***P***
Age, mean ± SD	74.5 ± 7.5	76.4 ± 7.2	0.0687
Gender (male), *N* (%)	46 (63.0)	41 (48.2)	0.078
Family status (married), *N* (%)	42 (57.5)	33 (38.8)	0.025
Living status (alone), *N* (%)	16 (21.9)	31 (36.5)	0.055
Nursing caregiver (yes), *N* (%)	28 (38.4)	31 (36.5)	0.870
Charlson's comorbidity index (Total score), mean ± SD	2.6 ± 1.9	2.2 ± 1.8	0.130
Dementia per history, *N* (%)	7 (9.6)	5 (5.9)	0.548
Medications during hospitalization, mean ±SD	6.7 ± 3.2	6.7 ± 2.8	0.911
Anticholinergic burden, mean ± SD	1.8 ± 1.5	1.9 ± 1.6	0.628
Pain (VAS>3)	0 (0.0)	3 (3.5)	0.249
Hemoglobin (g/dl), mean ± SD	11.8 ± 2.3	12.1 ± 2.2	0.334
Hyponatremia (Sodium <135 mEq/L)	8 (11.0)	13 (15.3)	0.486
Hypocalcemia (Calcium <8.5 mg/dl)	7 (9.6)	16 (18.8)	0.117
Delirium diagnosed by medical staff	7 (9.6)	4 (4.7)	0.348

### Health Outcomes in the CAM Group (Table 4)

#### SSD vs. No Delirium

Compared to the no delirium group fewer patients in this group were discharged to their home at the end of hospitalization (78.3 vs. 92.2%, *P* = 0.05) and the 3-month mortality rate was higher (26.1 vs. 8.7%, *P* = 0.03).

**Table 4:** Outcomes of the CAM group.

	**SSD (*N* = 23)**	**ASD (*N* = 31)**	**No D (*N* = 127)**	**P**	**P**
				**SSD vs. No D**	**ASD vs. No D**
Hospitalization time, mean ± SD	4.2 ± 5.8	4.8 ± 5.5	3.4 ± 4.0	0.635	0.102
Discharge, home	18 (78.3)	23 (74.2)	118 (92.2)	0.05	0.007
**Mortality rates**, ***N*** **(%)**					
During index hospitalization	0 (0.0)	0 (0.0)	2 (1.6)	0.382	0.488
90 days	6 (26.1)	10 (32.3)	11 (8.7)	0.03	0.002

*D, Delirium; ASD, any symptoms of delirium; SSD, Subclinical Delirium*.

#### ASD vs. No Delirium

Compared to the no delirium group fewer patients in the ASD group were discharged to their home at the end of hospitalization (74.2 vs. 92.2%, *P* = 0.007) and the 3-month mortality rate was higher with 10 deaths in the ASD group (32.3%) compared to 11 (8.7%) in the no delirium group (*P* = 0.002).

### Diagnosis of Delirium by the Medical Team in the CAM Group

The diagnosis of delirium in the CAM group (two experimental wards), or any other identical diagnosis such as acute confusion or confusional state, was found in the medical records of six of 31 patients with ASD (19.4%) and among 5 of 127 patients (3.9%) with no delirium. Compared to the diagnosis by the investigators, the sensitivity for the medical team was 19.4% and the specificity was 96.1%. Four of the eight patients in the full delirium (50%) and only two (8.7%) of the patients with SSD were diagnosed with delirium by the medical team (*P* = 0.028).

### Comparison Between the CAM Wards and the Control Ward (Table 5)

Only two of the 174 patients in the control ward had a diagnosis of delirium in their medical record, a much lower rate than in the CAM wards (1.2 vs. 7.0%, *P* = 0.007). The patients in the two groups (CAM vs. control wards) were similar in terms of age, sex, family status, living conditions, nursing caregiver, and the percentage of patients with a pre-admission diagnosis of dementia. The rates of infection or gastrointestinal problems as the reason for hospitalization was lower in the CAM group (20.3 vs. 31.6%, *P* = 0.02 and 5.7 vs. 12.1%, *P* = 0.045, respectively). In the CAM group the admission CRP level was lower (3.6 vs. 6.0 vs. 5.5 ± 7.7, *P* < 0.0001) and during the course of the hospital stay fewer patients had at least one occurrence of fever >38C (8.9 vs. 23.0%, *P* = 0.0004). In addition, in the CM group there were fewer cases of hypotension (5.1 vs.16.7%, *P* = 0.0007) tachycardia (16.5 vs. 32.2%, *P* = 0.0009) and pain greater than VAS>3 (7.0 vs. 20.7%, *P* = 0.0003).

**Table 5:** Comparison between CAM and control groups.

	**CAM group** ***N* = 158**	**Control group** ***N* = 174**	***P***
Delirium [based on charts, *N* (%)]	11 (7.0)	2 (1.2)	0.007
Age (years), mean ± SD	75.3 ± 7.4	76.3 ± 7.8	0.286
Gender (male), *N* (%)	87 (55.1)	90 (51.7)	0.545
Family status (married), *N* (%)	76 (48.1)	101 (58.1)	0.07
Living status (alone), *N* (%)	47 (29.7)	39 (22.4)	0.131
Nursing caregiver (yes), *N* (%)	59 (37.3)	82 (47.1)	0.073
Charlson's comorbidity index (Total score), mean ± SD	2.4 ± 1.8	2.8 ± 2.0	0.194
Dementia in anamnesis, *N* (%)	12 (7.6)	18 (10.4)	0.392
Medications during hospitalization, mean ± SD	6.7 ± 3.0	7.3 ± 3.0	0.083
AC Burden, *n* ± SD	1.8 ± 1.6	1.9 ± 1.6	0.814
**Reasons for hospitalization**, ***n*** **(%)**			
Any infection, *N* (%)	32 (20.3)	55 (31.6)	0.019
Cardio-vascular disease (IHD, HF, arrhythmia, CVA), *N* (%)	80 (50.6)	84 (48.3)	0.67
Gastro-intestinal disease, *N* (%)	9 (5.7)	21 (12.1)	0.045
COPD/Asthma, *N* (%)	18 (11.4)	17 (9.8)	0.635
Musculo-skeletal problems	21 (13.3)	16 (9.2)	0.178
**Lab on admission**, ***N*** **(%)**			
Hemoglobin(g/dl), mean ± SD	12.0 ± 2.2	11.6 ± 2.3	0.138
WBC (x10^3^ uL), mean ± SD	9.6 ± 7.0	9.2 ± 4.4	0.571
Glucose (mg/dl), mean ± SD	152.9 ± 78.0	145.3 ± 77.5	0.571
Creatinine (mg/dl), mean ± SD	1.3 ± 1.3	1.5 ± 1.4	0.698
Urea (mg/dl), mean ± SD	59.9 ± 47.8	63.5 ± 45.0	0.439
C-reactive protein, mean ± SD	3.6 ± 6.0	5.5 ± 7.7	<0.001
Hyponatremia (Sodium <135 mEq/L), *N* (%)	21 (13.3)	37 (21.3)	0.058
Hypernatremia (Sodium>145 mEq/L), *N* (%)	3 (1.9)	3 (1.7)	0.909
Hypocalcemia (Calcium <8.5 mg/dl), *N* (%)	23 (14.6)	31 (17.8)	0.427
Hypercalcemia (Ca >10.5 mg/dl), *N* (%)	2 (1.3)	1 (0.6)	0.571
**Signs during hospitalization**, ***N*** **(%)**			
Temperature≥38.0°C, *N* (%)	14 (8.9)	40 (23.0)	0.0004
Hypertension (Systolic BP ≥150 mmHg), *N* (%)	74 (46.9)	83 (47.7)	0.876
Hypotension (Systolic BP <90 mmHg), *N* (%)	8 (5.1)	29 (16.7)	0.0007
Tachycardia (HR> 100 per min), *N* (%)	26 (16.5)	56 (32.2)	0.0009
Bradycardia (HR <60 per min), *N* (%)	51 (32.3)	45 (25.9)	0.201
Pain (VAS>3), *N* (%)	11 (7.0)	36 (20.7)	0.0003

*AC, anticholinergic; BP, blood pressure; HR, heart rate; VAS, Visual Analog Scale; COPD, chronic obstructive pulmonary disease; CVA, cerebrovascular accident; IHD, ischemic heart disease; HF, heart failure; WBC, white blood cells*.

## Discussion

### Occurrence Rate of Delirium

The rate of occurrence of ASD during hospitalization was 19.6% with 5.1% full delirium and 14.6% SSD. These rates were on the low side of the range reported in other studies in the world (2–5). However, it is very likely that there are large differences among the studies in terms of the type of medical center, the composition of the hospitalized patients, and the selection of the study population. It is noteworthy that in our study we did not include patients with significant health issues, patients with severe dementia, or terminally ill patients. It is reasonable to assume that the patients in these groups would have a higher rate of delirium.

Another explanation for the relatively low rate of patients with delirium is that 20 of 23 patients with SSD lacked the criterion of acute onset and fluctuating course. None of the patients was accompanied by another person at the time of the CAM assessment and the medical team was not able to provide us with relevant information. Thus, it is reasonable to assume that at least some of the cases of SSD might actually have had full delirium. In Ward B where the CAM assessment was conducted only once, the rates of ASD and SSD were significantly higher than in Ward A where several CAM assessments were conducted. While the patients in Ward B tended to be older and had significantly fewer married patients, no differences were found in any of the other risk factors including clinical and laboratory variables. No differences were found between patients in the two wards, which are similar in composition and function in the same hospital. It is not likely that the difference is related to treatment or physical conditions in the ward. The same steps to prevent delirium were taken in both wards. It is possible that the number of patients was too small to have sufficient power to draw conclusions. In any case, the frequency of CAM assessments was not associated with an increase in the diagnostic rate for delirium.

### Risk Factors for Delirium

The identification of risk factors is critical for the identification of patients at risk, selection of the appropriate strategy, and close monitoring of patients (18).

We found that marriage was a protective factor for the development of ASD as well as SSD. Foroughan et al. (26) found that marriage reduces the risk for delirium among hospitalized patients. A study of patients hospitalized in an intensive care unit found that playing reassuring updated reorientation messages with a familiar voice led to a significant increase in delirium-free days (27). It is possible that the daily communication with a partner during the course of hospitalization contributes to the preservation of cognition as implemented in the program to prevent delirium (28).

The anticholinergic burden of drugs increased the risk for the development of ASD and SSD. The association between drugs with an anticholinergic burden and delirium has been reported in previous studies (29–31). Dementia was found to be a risk factor for ASD, as was reported in many previous studies that showed that impaired cognition (5, 8) and dementia (3, 32, 33) were risk factors for delirium.

The presence of a nursing caregiver prior to hospitalization was a risk factor for ASD, but not for SSD, whereas a low level of hemoglobin hypocalcemia, and a high pain score (Tables 1, 2) were statistically significant risk factors for ASD and SSD. In previous studies these factors were associated with delirium: functional impairment (3, 26, 34) pain (26, 35), low hemoglobin or hematocrit level (26, 34, 36), hyponatremia (37, 38), and hypocalcemia (32, 39). In studies of risk factors for SSD an association was found with some of the same factors that were significantly associated with it in the present study in univariate analyses, including functional impairment (7, 18), pain (40), and hyponatremia (18).

### Hospitalization Outcomes

A significantly lower percentage of patients with ASD and SSD were discharged from the hospital to their homes than with no delirium. Similar findings were reported in other studies including patients with delirium (3, 6, 41) and patients with SSD (7, 18, 41). Furthermore, there was a statistically significant increase in mortality over the 3-month follow-up in the ASD and SSD groups. The association of increased mortality with delirium is well-known (3, 6, 7). In the study by Zipprich et al. an association was reported between SSD and increased mortality (18). The authors concluded that SSD patients should receive the same clinical attention as patients with full delirium (18).

### Identification of Delirium by the Medical Team

We evaluated the sensitivity rate for the identification of delirium by whether the diagnosis was recorded in the medical record. In the CAM wards the rate was 19.4%, which is similar to the findings of systematic review by Barron and Holmes (12). This result is of concern since in the first phase of the study the medical teams in the experimental wards heard a lecture on delirium and over the course of the study the investigators were in the CAM wards for many hours and interacted closely with the teams, and each case of delirium was reported to the medical team.

In the control ward the medical team heard a lecture and received written material on delirium but did not interact with the study investigators. This could explain the large difference in the rate of diagnosis of delirium between the CAM wards and the control ward. Since the study participants in the control ward apparently were more “medically” complex, we anticipated similar rates for delirium in the medical records, but we found lower rates. This could lead to the assumption that the presence of an investigator in the ward who constantly reminded the medical team of the importance of identifying delirium had a positive effect on the diagnosis of delirium. Support for this contention can be seen in the results of a previous study conducted in an emergency department of the same medical center, which found that the presence of a specialist in geriatrics medicine increased the rate of diagnosis of delirium and cognitive assessments by the other doctors on the staff significantly (42). In a study by Davis and MacLullich (10) the investigators found that, for the most part, doctors had a good understanding of the importance of delirium, so the main barrier to effective coping with it was a lack of knowledge on its diagnosis and treatment.

Thus, there are good reasons to develop a comprehensive training program for relevant medical teams. Furthermore, there would appear to be a need for the introduction of structured screening methods, such as CAM, to the work routine in hospital wards to improve the rate of diagnosis of delirium (18).

### Study Strengths

To our knowledge this is the first prospective study that entailed a systematic evaluation of the rate of delirium in internal medicine departments of a big tertiary hospital in Israel.

### Study Limitations

This study has several limitations. First, only 44% of the overall patient sample in the CAM group was assessed for eligibility due to technical difficulties including lack of an available independent doctor, patients who spoke a foreign language only, patients away from the ward at the time, and the outbreak of the Corona virus, all of which made it difficult for the research team to recruit patients. On the one hand this left us with a relatively small sample size while on the other we do not know whether the patients that were not included in the study were different from those who did, which could have led to a selection bias.

Another significant limitation that is associated, at least in part, with the availability of the research staff is that some of the patients in Ward A did not undergo daily CAM assessments. Nineteen of the 73 patients in Ward A were discharged after only 1 day of hospitalization. The mean length of hospital stay for the other 54 patients was 4.4 days, or an additional 3.4 hospital days after the first CAM assessment. Since there were only about 70 repeat CAM assessments it is clear that a large proportion of the participants did not undergo repeat assessments. While the rates of ASD and SSD were higher in Ward B the low rate of repeat assessments in Ward A did not enable us to provide a definitive answer to one of the study questions, i.e., does a higher frequency of CAM assessment increase the diagnostic rate of delirium. In future studies investigators should take into account the difficulties that have been described here in terms of patient recruitment and difficulties related to research staff availability. For example, by increasing the number of researchers and defining the recruitment period that ended abruptly in the present study due to the Corona pandemic, and by strengthening ties between the research team and the staff in the experimental ward.

The study group was relative healthy compared to other patients hospitalized in the CAM wards, since severely ill patients such as patients with severe dementia, ventilated or terminally ill patients were excluded from the study. It is reasonable to assume that the rate of delirium would have been much higher in this group. All these factors limited our ability to extrapolate the results of the study to the general population of patients hospitalized in the internal medicine wards. Another significant limitation was that we didn't categorize the patients into different types of delirium; hyperactive; and hypoactive. In studies by Kiely et al. the investigators found that the type of delirium has a critical impact on health outcomes. They found, in a multivariate model, that among patients with delirium in post-acute care the hypoactive type, but not the mixed or hyperactive types of delirium, increased the risk of death in the first year after admission (43). In the present study the absence of information on the type of delirium prevented us from understanding some important aspects. For example, as noted above, the doctors were more capable of identifying full delirium than SSD, but it not clear whether the patients with full delirium were in a state of hyperactive delirium, which appears to be easier to diagnose.

There are several other notable limitations. Since the assessments were conducted during the morning and afternoon hours, patients with Sundowning were probably not included in the study. Some important factors for the development of delirium were not assessed (6), such as depression, an in-place urinary catheter, and visual and auditory impairment. Since we only used laboratory tests that were ordered by the medical team, we did not have albumin levels. Thus, we could not determine whether the association of hypocalcemia and delirium was independent of associated with a low protein level.

We used the Charlson's Comorbidity Index to assess comorbidity. This index evaluates the number of diseases, but not their severity, although there are some exceptions to this including diabetes mellitus and liver disease. This may be the reason that this index was not included as a risk factor for the development of delirium. Future studies should consider whether to use a comorbidity score that also assesses disease severity.

Having a nursing caregiver reflects the patient's functional status prior to hospitalization, but it is important to note that the presence of a nursing caregiver reflects not only functional status, but the willingness of the patient to try to preserve an independent lifestyle despite functional impairment. Thus, the absence of a direct measure of baseline function is an additional limitation of this study. Finally, we note that in this study the follow-up for morality continued for 90 days after admission to the hospital, so we still do not have a long-range follow-up. We are planning to continue following the study participants for a year to see if delirium patients had different health-related behavior (repeat hospitalization, emergency room visits, utilization of healthcare services).

In summary, the present study provides further proof that delirium, including SSD, is not a rare problem among hospitalized patients in internal medicine departments.

Delirium, as well as SSD, have significant prognostic importance in terms of discharge from the hospital and mortality. However, most cases or delirium are not diagnosed by the medical team. There is need for an intervention program that would include more comprehensive training for the medical team and the introduction of CAM as a routine test in medical wards.

## Data Availability Statement

The raw data supporting the conclusions of this article will be made available by the authors, without undue reservation.

## Ethics Statement

The studies involving human participants were reviewed and approved by Helsinki Committee of the Soroka Medical Center (SOR-0047-19). Written informed consent for participation was not required for this study in accordance with the national legislation and the institutional requirements.

## Author Contributions

NE designed the study, collected the data, and wrote the article. EZ, DS, DK, and EM designed the study, collected the data, and assisted with wrote the article. LB, LZ, and CB designed the study and assisted with wrote the article. TF designed the study, was responsible for the statistical design of the study, and for carrying out the statistical analysis. SL designed the study collected the data and assisted with wrote the article. YP designed the study, collected the data, and wrote the article. All authors contributed to the article and approved the submitted version.

## Conflict of Interest

The authors declare that the research was conducted in the absence of any commercial or financial relationships that could be construed as a potential conflict of interest.
